# State of Mental Health and Associated Factors in Nursing Students from Southeastern Iran

**DOI:** 10.17533/udea.iee.v37n3e04

**Published:** 2019-10-23

**Authors:** Zinat Mohebbi, Giti Setoodeh, Camelia Torabizadeh, Masoume Rambod

**Affiliations:** 1 Ph.D. Department of Medical Surgical Nursing, Shiraz University of Medical Sciences, Shiraz, Iran. Email: mohebbi04@yahoo.com Corresponding author. Shiraz University of Medical Sciences Shiraz Iran mohebbi04@yahoo.com; 2 Ph.D. Candidate. Department of Psychiatric Nursing, Shiraz University of Medical Sciences, Shiraz, Iran. Email: setoodeh@sums.ac.ir Shiraz University of Medical Sciences Shiraz Iran setoodeh@sums.ac.ir; 3 Ph.D. Department of Medical Surgical Nursing, Shiraz University of Medical Sciences, Shiraz, Iran. Shiraz University of Medical Sciences Shiraz Iran; 4 Ph.D. Community Based Psychiatric Care Research Center, Shiraz University of Medical Sciences, Shiraz, Iran. Shiraz University of Medical Sciences Shiraz Iran

**Keywords:** students, nursing, mental health, depression, anxiety, sleep wake disorders, confounding factors (epidemiology), surveys and questionnaires., estudiantes de enfermería, salud mental, depresión, ansiedad, trastornos del sueño-vigilia, factores de confusión (epidemiología), encuestas y cuestionarios., estudantes de enfermagem, saúde mental, depressão, ansiedade, transtornos do sono-vigília, fatores de confusão (epidemiologia), inquéritos e questionários.

## Abstract

**Objective.:**

To evaluate the state of mental health and its relation with associated factors among nursing students.

**Methods.:**

A cross-sectional study was conducted with 130 students from the Nursing and Midwifery College affiliated to the University of Medical Sciences of Shiraz (Iran). Data was collected through a document that included information on the demographic characteristics, the mean grades of the practical assignments and of the total (practical and theoretical assignments), and the Goldberg Health Questionnaire (GHQ-28) that measures symptoms grouped into four dimensions (somatic symptoms, anxiety and insomnia, social dysfunction, and depression).

**Results.:**

Most of the participants (65.1%) were women; 5.3% were between 21 and 22 years of age, 84.5% were single, and 33.3% were in the sixth semester; 68.5% of the students had problems with mental health. By dimensions of the GHQ-28, it was found that 7.7% had somatic symptoms, 13.8% symptoms of anxiety and sleep disorders, 52.3% social dysfunction, and 6.2% depression. Males had a higher score of depression than females, and being single was related with higher scores of physical symptoms, anxiety and insomnia, and depression, compared with those who were married. An inverse relationship was found between the GHQ-28 average score and the semester, the grade in practical assignments, and the total grade for physical symptoms and anxiety and insomnia.

**Conclusion.:**

There is a high proportion of nursing students with suspected mental health disorder. Some demographic and academic factors are related with the mental health of students and must be kept in mind by the institutions training future nurses.

## Resumen

**Objetivo.:**

Evaluar el estado de salud mental y su relación con factores asociados entre los estudiantes de enfermería.

**Métodos.:**

Se realizó un estudio de corte transversal con 130 estudiantes del Colegio de Enfermería y Partería afiliado a la Universidad de Ciencias Médicas de Shiraz (Irán). Para la toma de los datos se empleó un formulario que incluía información sobre las características demográficas, los promedios de calificación de las asignaturas prácticas y del total (asignaturas prácticas más teóricas), y el Cuestionario de Salud Goldberg (GHQ-28) que mide síntomas agrupados en cuatro dimensiones (síntomas somáticos, ansiedad e insomnio, disfunción social, y depresión).

**Resultados.:**

La mayoría de los participantes (65.1%) fueron mujeres; el 5.3% tenía entre 21 y 22 años; solteros, el 84.5% y el 33.3% estudiaba en el sexto semestre. El 68.5% de los estudiantes tenía problemas con la salud mental. Por dimensiones del GHQ-28, se encontró que el 7.7% tenía síntomas somáticos; 13.8%, síntomas de ansiedad y trastornos del sueño; 52.3%, disfunción social y 6.2% depresión. El sexo masculino tuvo mayor puntaje de depresión que las mujeres, y el estado civil soltero se relacionó con mayores puntajes de síntomas físicos, ansiedad e insomnio y depresión, comparado con los casados. Se encontró una relación inversa entre el promedio del puntaje del GHQ-28 y el semestre, la calificación en las asignaturas prácticas y la calificación total para síntomas físicos y ansiedad e insomnio.

**Conclusión.:**

Es alta la proporción de estudiantes de enfermería con sospecha de trastorno de la salud mental. Algunos factores demográficos y académicos están relacionados con su salud mental y deben ser tenidos en cuenta por las instituciones formadoras de los futuros enfermeros.

## Resumo

**Objetivo.:**

Avaliar o estado de saúde mental e sua relação com fatores associados entre os estudantes de enfermagem.

**Métodos.:**

Se realizou um estudo de corte transversal com 130 estudantes do Colégio de Enfermagem e Parteira afiliada à Universidade de Ciências Médicas de Shiraz (Irã). Para a toma dos dados se empregou um formulário que incluía informação sobre as características demográficas, as médias de qualificação das matérias práticas e do total (matérias práticas mais teóricas), e o Questionário de Saúde Goldberg (GHQ-28) que mede sintomas agrupados em quatro dimensões (sintomas somáticos, ansiedade e insônia, disfunção social, e depressão).

**Resultados.:**

A maioria dos participantes (65.1%) eram mulheres; 5.3% tinham entre 21 e 22 anos, eram solteiros (84.5%) e o 33.3% estudavam no sexto semestre. 68.5% dos estudantes tinham problemas com a saúde mental. Por dimensões de GHQ-28, se encontrou que 7.7% tinham sintomas somáticos, 13.8% sintomas de ansiedade e transtornos do sono, 52.3% disfunção social e 6.2% depressão. O sexo masculino teve maior pontuação de depressão que as mulheres, e o estado civil solteiro se relacionou com maiores pontuações de sintomas físicos, ansiedade e insônia e depressão, comparado com os casados. Se encontrou uma relação inversa entre a média da pontuação de GHQ-28 e o semestre, a qualificação nas matérias práticas e a qualificação total para sintomas físicos e ansiedade e insônia.

**Conclusão.:**

É alta a proporção de estudantes de enfermagem com suspeita de transtorno da saúde mental. Alguns fatores demográficos e acadêmicos estão relacionados com a saúde mental dos estudantes e devem ser tidos em conta pelas instituições formadoras dos futuros enfermeiros.

## Introduction

Mental health is defined as a state of well-being in which individuals realize their own potential, can cope with normal stresses of life, can work productively and fruitfully, and are able to contribute to their community.(1) According to Kamau,(2) a person enjoying mental health is one who is away from anxiety and disability symptoms, can communicate well with others, and is able to face life’s pressure. According to another theory, mental health is “the adaptation of individuals with their surrounding world to the extent that it causes happiness and useful and effective enjoyment completely”.(3) Actually, a person with good mental health has a better function and quality of life, compared with others.(4) Mental health is a subject which has been studied vastly among students during recent years. Health problems are numerous and increase among students.(5) The importance of studying mental health in universities is valuable because it could have direct relation with the educational progress of students.(6) Student life, non-acquaintance with the new educational environment, remaining away from family, being uninterested with the educational field, incompatibility with others in the student life environment, socio-economic problems, and the lack of welfare facilities are among the causes creating mental problems and inconveniences and, finally, causes educational subsidence.(7,8)

The prevalence of mental disorder among students has been addressed by researchers. In the research carried out, this rate has been reported at 50.0% and 30.0% among students from universities in the USA and Europe, respectively.(8,9) According to such reports, 79.2% of students from African universities and 43.2% from Asian universities also have symptoms of mental disorder.(6,10) Also, according to a study conducted, the rate of depression disorder in students from Iranian universities is reported at 28.04%.(11) Considering the importance of the mental health of students, specially nursing students, due to the nature of this educational field and because they have direct contact with patients and their relatives and other health staff and that no research has been carried out in this respect among nursing students from Shiraz University and paying attention to the fact that, general health is such an index that should be measured in various individuals of a society during different periods of life; therefore, researchers decided to carry out a study to determine the mental health status among such students. This will be the first step to promote the level their mental health, so that students with this kind of problems would be identified for diagnosis and treatment and, in this way, complications in their mental health state and poor educational performance would be prevented.

## Methods

A descriptive, cross-sectional study was conducted between 2016 and 2017. The variable of mental health of undergraduate nursing students was evaluated in this study. All undergraduate nursing students studying at the Nursing and Midwifery College affiliated to the Shiraz University of Medical Sciences comprised the research population and all accessible students who had propensity of participating in the study and signed the informed consent were evaluated. Of the 176 students selected, only 130 delivered the questionnaire completely filled out to the researchers. Data collecting tools consisted of demographic characteristics (sex, age, marriage status), academic characteristics (semester of study, training score and total average and Goldberg Health Questionnaire (GHQ-28) to measure general health. The training score means the grade of practical lessons and the average is the total average of theoretical and practical assignments. This questionnaire includes 28 multiple choice questions used by Goldberg and Hiler in 1972 to recognize the mild mental disorder.(12) The questions in this questionnaire include four levels, namely, “much lower than regular level with the score of zero”, “lower than regular level with the score of one”, “at the regular level with the score of two” and “more than regular level with the score of three” and its score range will be from 0 to 84. Each dimension consists of seven questions and the maximum score in each dimension is 21 and in total, there are 84 scores and the higher score specifies lower general health.(13) This tool included four sub-scales in social function, anxiety and sleep disorder, depression, and disorder in physical health. The cutoff point of 23.0 and 14.0 was considered to determine mental health disorder and for the sub-scale, respectively. The validity as well as reliability of the Persian version of questionnaire was confirmed in various studies.(14,15) The reliability coefficient for the whole GHQ was 0.96 and for the sub-scales of depression, anxiety, physical and social disorder, it was determined as 0.94, 0.90, 0.89, and 0.7, respectively.(16) Cronbachʼs alpha in this research was calculated at 0.85. Data collected were analyzed by using SPSS version 23 software, with help from descriptive statistic tests, independent tests, Kruskal-Wallis H, Mann-Whitney U, and One Way Analysis of Variance. The statistical significance level for the tests was 0.05.

## Results

In this study, most of the participants (65.1%) were women, with mean age ranging from 21 to 22 years (55.3%). Most of students (84.5%) were single and 33.3% of them were in the 6th semester. The maximum training score of the participants was 19.0 and the minimum was 15.5; the mean of the total average of all the students was 15.62±1.62. In this investigation, only 30.0% of students were healthy and 68.5% of them were suspected of suffering from mental health disorder (Table 1). By evaluating each questionnaire domain, it was specified that 7.7%, 13.8%, 52.3%, and 6.2% of the individuals suffered from disorders in the physical dimension, symptoms of anxiety and sleep disorder, disorder in social function, and symptoms of depression, respectively. The most prevalent disorder was observed in social function (Table 1).

**Table 1:** Frequency of healthy individuals and those suspected of suffering from mental health disorder (*n* = 130)

Mental Health	Healthy	Healthy	Doubtful about disorder	Doubtful about disorder	Missing data	Missing data
	Number	Percentage	Number	Percentage	Number	Percentage
Total score of GH	39	30.0	89	68.5	2	1.5
Physical symptoms	116	89.2	10	7.7	4	3.1
Symptoms of anxiety and sleep disorder	108	83.1	18	13.8	4	3.1
Social Dysfunction	56	43.1	68	52.3	6	4.8
Symptoms of depression	119	91.5	8	6.2	3	2.3

Table 2 shows the average scores of the total and by domains of the GHQ-28, according to demographic variables. Males had higher average scores, exclusively in the domain of depression; and those who were single in the scale total and in the domains of physical symptoms, anxiety and insomnia, and depression. Age was not significantly related with the scale score in any domain or with the total.

**Table 2:** Mean and standard deviation of the scores of four domains of mental health according to demographic characteristics

Dimensions	Variables	Physical symptoms	Anxiety and Insomnia	Social dysfunction	Depression	Total scale
Sex	Male	5.88±3.77	6.88±4.79	13.39±3.48	5.37±5.50	30.50±10.34
	Female	6.96±4.53	7.01±5.86	13.06±3.89	2.79±3.98	29.34±10.55
	Test	t = 1.34 *p* = 0.18	t = 0.12 *p* = 0.90	Z = 0.33 *p* = 0.74	Z = 2.76 *p* = 0.006	t = 0.59 *p* = 0.55
Age	19-20	7.24±5.11	7.43±5.71	12.66±4.26	4.13±4.91	30.31±11.65
	21-22	6.77±4.04	6.74±5.59	13.37±3.53	3.47±4.62	29.77±10.22
	23-29	4.18±3.12	5.87±4.41	13.93±3.67	3.62±5.26	27.62±9.81
	Test	F = 2.98 *p* = 0.055	F = 0.47 *p* = 0.62	F = 0.72 *p* = 0.48	X2 = 1.009 *p* = 0.63	F = 0.36 *p* = 0.69
Marriage Status	Single	6.94±4.48	7.36±5.58	12.95±3.91	4.18±4.87	30.79±10.79
	Married	4.38±2.06	4.50±4.42	14.63±2.11	1.52±1.02	23.57±5.60
	Test	F = 49.82 *p* <0.001	t = 2.067 *p* = 0.04	Z = -1.62 *p* = 0.105	Z = -3.89 *p* <0.001	t = 4.36 *p* = 0.001

For the academic variables, the average of the GHQ-28 noted that the significant and inverse relation between educational semester and the score of the depression domain could be mentioned as the other results of this research in such a way that students in higher semesters obtained a lower depression score. The training score showed a significant and also inverse relationship with the global score, as well as the score of physical symptoms and anxiety domains; meaning that those who had a better training scores obtained a lower score of mental health, physical symptoms, and anxiety. The total average score had a borderline statistical significance with the scores of the total scale and its domains (Table 3).

**Table 3:** Mean and standard deviation of the scores of four domains of mental health according to the educational semester, training score, and total average

Variables	Dimensions	Physical symptoms	Anxiety and insomnia	Social dysfunction	Depression	Total scale
Educational Semester	3	60.50±4.18	8.09±5.67	12.93±3.18	5.67±5.41	32.21±11.63
	4	7.55±4.88	7.39±6.08	13.14±4.60	3.40±5.05	30.85±10.96
	6	6.32±3.88	5.79±4.28	13.14±3.34	3.02±4.05	27.67±8.92
	7	5.47±4.24	7.00±6.47	13.66±3.94	2.27±3.02	28.11±10.23
	Test	F=1.01 *p*=0.38	F=1.17 *p*=0.32	X2=0.82 *p*=0.84	X2=8.11 *p*=0.04	F=1.45 *p*=0.23
Training score	11-14	8.38±4.38	9.19±5.60	12.20±4.06	5.28±5.61	33.92±11.65
	14.01-16	6.30±4.05	5.60±4.95	13.44±3.96	3.10±4.24	27.97±9.78
	16.01-20	5.86±3.82	6.05±4.98	12.97±3.03	3.76±4.97	28.31±9.59
	Test	F = 3.21 *p* = 0.044	F = 4.23 *p* = 0.017	X2 = 2.40 *p* = 0.30	X2 = 1.68 *p* = 0.43	F = 3.12 *p* = 0.048
Total average	11-14	6.30±3.63	6.91±4.89	14.31±3.02	5.21±5.71	31.08±9.85
	14.01-16	5.95±4.09	5.93±5.18	13.72±3.59	2.80±3.95	27.43±10.46
	16.01-20	6.77±4.24	7.30±5.50	12.50±3.73	3.28±3.97	29.86±9.12
	Test	f = 0.49 *p* = 0.61	f = 0.83 *p* = 0.43	X2 = 5.63 *p* = 0.06	X = 2.56 *p* = 0.27	f = 1.31 *p* = 0.27

## Discussion

In this study, only 30.0% of students had good mental health and the total score average of mental health of the Shiraz nursing students was reported at 29.74±10.45. A study in China in this respect in 2012 showed the general health of students at 22.9%.(17) A study in Sweden reported the prevalence of depression disorder among first-year nursing students at 16.4%(18) and 24.0% of medical students from the USA were suffering from depression.(19) According to a five-year prospective study in England, it was specified that 66.2% of medical students were suffering from at least one of the indexes of general health status. Also, the general health status of first-year students was accounted as a criterion for anticipating their mental health status during the coming years.(20) Considering the rate of mental health obtained, using the psychological and therapeutic methods, it seems necessary to improve the general health status of nursing students.

The results show that social function disorder was the most prevalent (52.3%) problem of students, which is similar to previous studies(20,21); thereby, more attention should be paid to studying the social function of students during student consultations.Students could be encouraged to have the best function, causing the support of their self-reliance by arranging the consultation and self-adjustment classes. Using the educational guides to support the social skills of students also seems necessary. The dimension of physical symptoms in the GHQ is an indication of the chance for individuals to suffer from physical illness.(22) In this study, the mean of 6.58±4.29 with 7.7% was obtained in this dimension. In this respect, Dalal and Bala(23) showed a mean of 23.7%.

In the depression domain, the lowest percentage (6.2%) with the mean of 3.66±4.69 was obtained. This dimension was mentioned as 2.7% in previous studies.(23) The American National Institute of Mental Health reported the rate of depression of students at 30.0%.(24) From the view point of the anxiety domain in our study, the rate of 13.8% with the mean of 6.96±5.49 was obtained. This index has been reported in other studies at 53.3%.(23) Suitable educational environment, good contact with professors and reciprocal understanding of students and professors could be effective in reducing such condition. Given that anxiety could affect other important aspects of life, causing disorder in the social function, paying attention to such is of great importance.

In this study the score of depression dimension of male students was higher than in female students. Most studies either did not obtain any statistical significant relation between women and men(25) or obtained significant statistical difference from the point of view of sex among students using alcohol, drugs, or tobacco.(26) It seems that, this lack of similarity is due to the existence of various reasons, like differentiating of cut-off point among sub-divisions in the present study, that nursing students were only the under study group while in the mentions studies.

The relation between marital status and the score of general health and sub-divisions of depression, physical symptoms, and anxiety was statistically significant in such a way that married people obtained better scores. In this respect, these findings were similar to research carried out in Mazandaran, Zahedan, Jiroft, and Iran University of Medical Sciences,(19,20) but are not similar to some studies.(24) This variation is likely due to the difference between the groups studied because none of the studies mentioned have been carried out with nursing students. The significant relation between semester of study and the score of the depression dimension could be mentioned as the other results of this research so that the students from higher semesters obtained a lower depression score. In this respect, the results of the study by Dalal and Bala showed that the general health of students from higher semesters is better than those in lower semesters.(23) Davidson also reported that anxiety among beginner students is more than that of seniors.(27) It seems that nursing students during the initial semesters, due to not being aquainted with the ward and with the nature of their educational field (most of the subjects are covered at the hospital and therapeutic centers), will adapt to the environment during the higher semesters and obtain better mental health.

The training score showed statistically significant relation with the score of mental health, as well as the score of the physical symptoms and anxiety sub-divisions, meaning that those who had better training scores obtained better scores in mental health, physical symptoms, and anxiety. In this respect, the results of this study were similar to the results from other studies.(21,23,25) In this study, the average score showed no statistically significant relation with the scores of mental health and its sub-divisions, but some of the studies mentioned showed reverse statistical relation between the mean score and the score of general health, meaning that higher mean score indicates lower score of general health so that the student’s mental status was better.

The conclusion of this research reveals that cases suspected of general health disorder among students from Shiraz University of Medical Sciences are comparatively high. Although the GHQ-28 cannot prove the physical or mental problems of students definitely, it recognizes students exposed to risk to some extent. University students report educational-related and general stressors in Iran.(28) This study also found that some demographic and academic factors are related with the mental health of the students, this is why they should be kept in mind by institutions training future nurses.

The limitations of this research could also interfere in explaining the results; refusal by some students to answer some questions and the small sample size are among the limitations of this study. No motivation or desire to answer the research questionnaire is the current problem of such studies. It seems that carrying out longitudinal research with a large sample size and considering the interfering variables and controlling the confounding variables is the most suitable choice to reach better results. Therefore, more specific evaluations to study this disorder could also determine the cause of the increase in this index compared with other studies carried out. Of course, by recognizing such students and arranging consultation classes with psychologists and psychiatrists, the risk factors could be reduced in these students.
